# The Age-Sensitive Efficacy of Calorie Restriction on Mitochondrial Biogenesis and mtDNA Damage in Rat Liver

**DOI:** 10.3390/ijms22041665

**Published:** 2021-02-07

**Authors:** Guglielmina Chimienti, Anna Picca, Flavio Fracasso, Francesco Russo, Antonella Orlando, Giuseppe Riezzo, Christiaan Leeuwenburgh, Vito Pesce, Angela Maria Serena Lezza

**Affiliations:** 1 Department of Biosciences, Biotechnologies and Biopharmaceutics, University of Bari Aldo Moro, Via Orabona 4, 70125 Bari, Italy; guglielminaalessandra.chimienti@uniba.it (G.C.); flavio.fracasso@uniba.it (F.F.); vito.pesce@uniba.it (V.P.); 2 Fondazione Policlinico Universitario “Agostino Gemelli” IRCCS, L.go F. Vito 8, 00168 Rome, Italy; anna.picca1@gmail.com; 3 Aging Research Center, Department of Neurobiology, Care Sciences and Society, Karolinska Institutet and Stockholm University, 11330 Stockholm, Sweden; 4 Laboratory of Nutritional Pathophysiology, National Institute of Gastroenterology “S. de Bellis”, Research Hospital, 70013 Castellana Grotte, Italy; francesco.russo@irccsdebellis.it (F.R.); antonella.orlando@irccsdebellis.it (A.O.); giuseppe.riezzo@irccsdebellis.it (G.R.); 5 Department of Aging and Geriatric Research, Institute on Aging, Division of Biology of Aging, University of Florida, Gainesville, FL 32611, USA; cleeuwen@ufl.edu

**Keywords:** rat liver, aging, calorie restriction, mitochondrial biogenesis, mtDNA damage, age-sensitive efficacy of CR

## Abstract

Calorie restriction (CR) is the most efficacious treatment to delay the onset of age-related changes such as mitochondrial dysfunction. However, the sensitivity of mitochondrial markers to CR and the age-related boundaries of CR efficacy are not fully elucidated. We used liver samples from ad libitum-fed (AL) rats divided in: 18-month-old (AL-18), 28-month-old (AL-28), and 32-month-old (AL-32) groups, and from CR-treated (CR) 28-month-old (CR-28) and 32-month-old (CR-32) counterparts to assay the effect of CR on several mitochondrial markers. The age-related decreases in citrate synthase activity, in TFAM, MFN2, and DRP1 protein amounts and in the mtDNA content in the AL-28 group were prevented in CR-28 counterparts. Accordingly, CR reduced oxidative mtDNA damage assessed through the incidence of oxidized purines at specific mtDNA regions in CR-28 animals. These findings support the anti-aging effect of CR up to 28 months. Conversely, the protein amounts of LonP1, Cyt c, OGG1, and APE1 and the 4.8 Kb mtDNA deletion content were not affected in CR-28 rats. The absence of significant differences between the AL-32 values and the CR-32 counterparts suggests an age-related boundary of CR efficacy at this age. However, this only partially curtails the CR benefits in counteracting the generalized aging decline and the related mitochondrial involvement.

## 1. Introduction

According to the impressive progression of studies investigating the process of aging, several different definitions of the process have sequentially been composed, and a set of pathways and mediators has been identified as the so-called hallmarks of aging [1,2]. Among these, mitochondrial dysfunction, oxidative stress, and molecular damage of mitochondria have emerged as a crucial factor. An important feature of aging is tissue-specificity that has been highlighted for mitochondrial involvement [3,4], epigenetic changes [5], and other alterations such as telomere length [6]. Among the various treatments aiming at preventing or delaying the onset of age-related changes, calorie restriction (CR) is the oldest strategy proposed [7], and still represents the gold standard for the very large number of beneficial effects conveyed by this intervention. Indeed, recent studies on the molecular mechanisms evoked by CR have demonstrated that several pathways are positively influenced by this nutritional intervention [8,9,10]. Furthermore, these findings also strongly support the existence of a “plasticity” of aging [5,9,11,12] that can be modulated via environmental interactions as nutritional or pharmaceutical interventions or life-style habits. CR has already been shown to counteract the age-related alterations of mitochondrial biogenesis and metabolism in several rat tissues with tissue-specific effects [3,13,14]. More specifically, previous studies by our group on rat liver, which is deeply affected by aging at the mitochondrial level, examining organellar markers showed a positive effect of CR on mitochondrial age-related alterations. In fact, in CR-treated aged 28-month-old rats, the mitochondrial DNA (mtDNA) content, amount of proteins involved in mitochondrial biogenesis or ROS-sensitive, and the extent of mitochondrial transcription factor A (TFAM) binding at specific mtDNA regions presented values significantly different from those of the age-matched ad libitum-fed (AL) counterparts and not statistically different from those of younger 18-month-old AL-fed animals [15]. More recently, the analysis of mitochondrial biogenesis and mtDNA damage in liver from AL aged (28-month-old) and extremely aged (32-month-old) rats, allowed us to study a diverse array of mitochondrial parameters and to suggest a different pace of aging in relation to the mitochondrial involvement, in the few animals naturally reaching longevity with respect to the majority of those becoming aged [16,17]. With these premises, we aimed at assessing the CR efficacy on a large set of mitochondrial markers at various ages in rat liver. For these purposes, we used animals from the very long-living hybrid strain Fischer 344 x Brown Norway, from which the 18-month-old ad libitum-fed rats (AL-18) were chosen as controls, to be compared with the ad libitum-fed, aged (AL-28) and extremely aged (AL-32) animals and the CR-treated age-matched counterparts (CR-28 and CR-32). In these groups of rats, we evaluated: The activity of the mitochondrial enzyme citrate synthase, the expression of a set of proteins including TFAM, Lon protease (LonP1) and cytochrome c (Cyt c), the mtDNA repair enzymes 8-oxoG DNA glycosylase/apurinic or apyrimidinic (AP) lyase (OGG1) and apurinic/apyrimidinic endonuclease 1 (APE1), and the mitochondrial dynamics proteins mitofusin 2 (MFN2) and dynamin-related protein 1 (DRP1). Finally, the mtDNA content and oxidative damage at specific regions were also determined.

## 2. Results

### 2.1. Evaluation of Age and CR Effects on Markers of Mitochondrial Functionality

**Figure 1:**
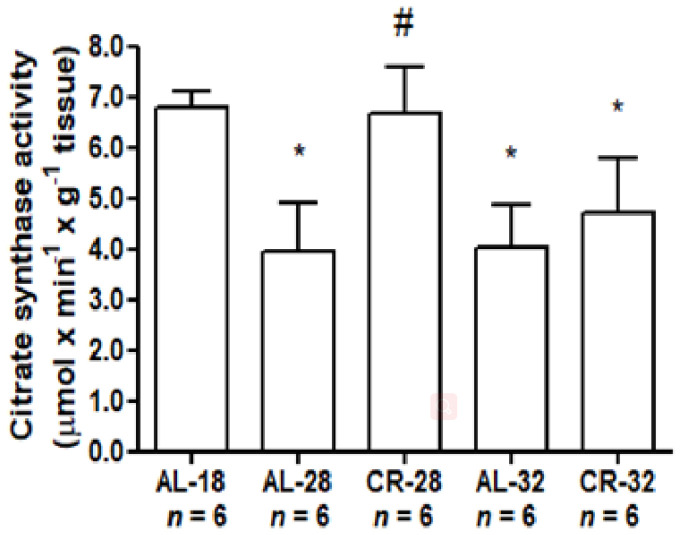
Citrate synthase activity in liver from AL-18, AL-28, CR-28, AL-32, and CR-32-month-old rats. Bars represent mean values and standard deviation (SD) of data obtained from experiments performed in quadruplicate and analyzed using the One-way ANOVA test and Tukey’s multiple comparison test. * *p* < 0.05 versus AL-18 rats, # *p* < 0.05 versus AL-28 rats. *n*: Number of analyzed animals.

The citrate synthase activity, which is also a marker of mitochondrial mass, was determined in all five groups of rats to evaluate the sensitivity of mitochondrial functionality to aging and CR (Figure 1).

An age-related statistically significant decrease in citrate synthase activity was observed in AL-28 (−41%) and AL-32 (−40%) rats compared to the AL-18 age control. No statistically significant difference was found between AL-28 and AL-32 groups. The CR effect was demonstrated by the significantly higher (+40%) enzyme activity in the CR-28 rats compared to the age-matched AL-28 rats value. Furthermore, the CR-28 activity was similar to that observed in the younger AL-18 control rats (Figure 1). This effect was instead blunted in the CR-32 animals. In fact, at the older age of 32 months no statistical difference could be observed between the AL and CR groups.

**Figure 2:**
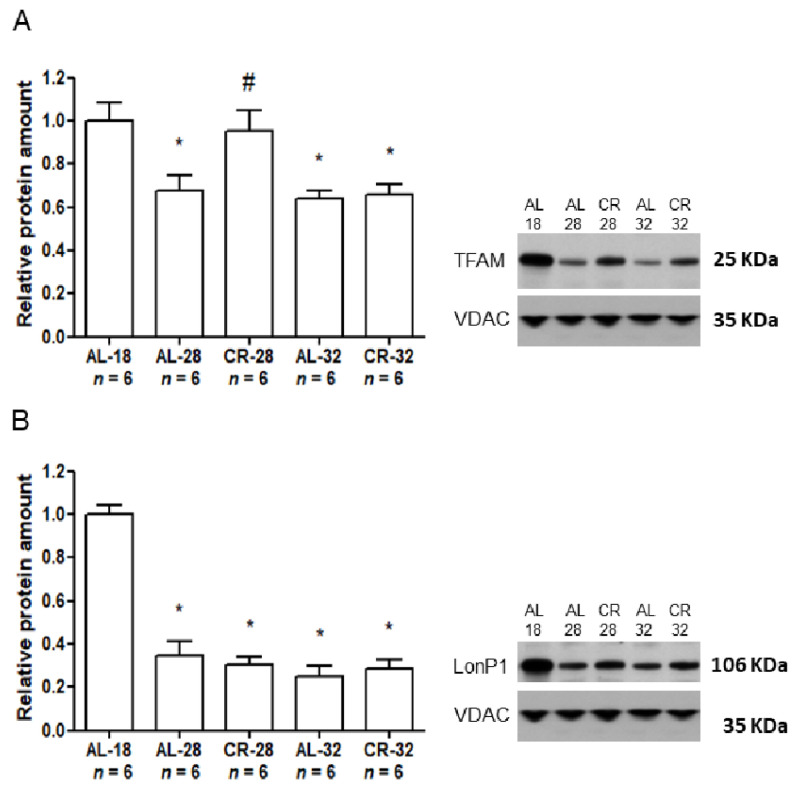
Amounts of TFAM and LonP1 proteins in isolated liver mitochondria from AL-18, AL-28, CR-28, AL-32, and CR-32-month-old rats. (**A**). TFAM protein amounts in isolated liver mitochondria from different rat groups. In the inset, a representative Western blot was carried out in one rat from each of the assayed groups. Immunoreactive bands, from top to bottom, represent, respectively, the signals from TFAM and VDAC. The histogram shows the quantification of the intensity of the bands of TFAM, normalized to the intensity of VDAC. (**B**). LonP1 protein amounts in isolated liver mitochondria from different rat groups. In the inset, a representative Western blot was carried out in one rat from each of the assayed groups. Immunoreactive bands, from top to bottom, represent, respectively, the signals from LonP1 and VDAC. The histogram shows the quantification of the intensity of the bands of LonP1, normalized to the intensity of VDAC. (**A**,**B**) Data represent the results from triplicate Western blot experiments and were analyzed using the One-way ANOVA test and Tukey’s multiple comparison test. Bars represent the mean values and SD for the five experimental groups. Data were normalized against the value of the AL-18 rats, fixed as 1. * *p* < 0.05 versus AL-18 rats, # *p* < 0.05 versus AL-28 rats. *n*: Number of analyzed animals.

To deepen the analysis of age and CR effects on mitochondrial functionality, the protein amount of TFAM and of LonP1 was determined in the mitochondria isolated from all rat groups by Western blot analyses, in which the protein from the outer mitochondrial membrane VDAC was used as an internal loading control (Figure 2).

Aging led to a statistically significant (−31%) decrease in the TFAM amount comparing AL-18 control rats with both AL-28 and AL-32 animals, whereas there was no significant difference between the values of AL-28 and AL-32 groups. As an effect of CR, the CR-28 rats had a TFAM amount significantly higher (+39%) than that of the age-matched AL counterparts and not different from that of the younger AL-18 controls. Conversely, such diet effect was not visible anymore in the CR-32 animals that showed a TFAM amount not statistically different from that of the age-matched AL group and significantly lower than that of the AL-18 rats. As for the LonP1 amount, there was a statistically significant (−65%) age-related decrease in the AL-28 group with respect to the AL-18 value. The age progression made the statistically significant decrease in the LonP1 amount, with respect to the AL-18 rats, more marked (−75%) in the AL-32 group. Interestingly, there was no evident effect of CR on the LonP1 amount since the comparison between the AL-28 value and the CR age-matched counterpart did not show any significant difference and the same occurred for the comparison between the AL-32 and CR-32 groups.

**Figure 3:**
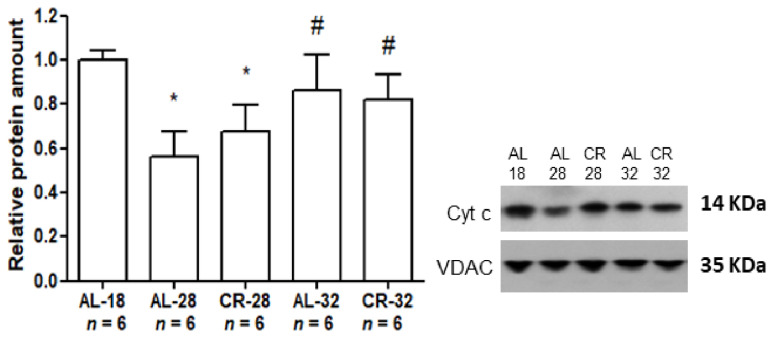
Amounts of Cyt c protein in isolated liver mitochondria from AL-18, AL-28, CR-28, AL-32, and CR-32-month-old rats. In the inset, a representative Western blot was carried out in one rat from each of the assayed groups. Immunoreactive bands, from top to bottom, represent, respectively, the signals from Cyt c and VDAC. The histogram shows the quantification of the intensity of the bands of Cyt c normalized to the intensity of VDAC. All data represent the results from triplicate Western blot experiments and were analyzed using the One-way ANOVA test and Tukey’s multiple comparison test. Bars represent the mean values and SD for the five experimental groups. Data were normalized against the value of the AL-18 rats, fixed as 1. * *p* < 0.05 versus AL-18 rats, # *p* < 0.05 versus AL-28 rats. *n*: Number of analyzed animals.

To evaluate the effect of aging and CR on another mitochondrial metabolic function which is the initiation of the mitochondrial intrinsic apoptotic pathway, we determined, by Western blot experiments, the intra-mitochondrial amount of Cyt c (Figure 3).

The age-related effect on the intra-mitochondrial Cyt c amount was peculiar since there was a marked (−44%) statistically significant decrease passing from the AL-18 rats to the AL-28 animals, but such decrease was partially prevented in the AL-32 group showing a small (−13%) decrease in comparison with the AL-18 counterpart and a significant marked (+55%) increase in comparison with the AL-28 rats. The CR effect was not relevant at all since there were no significant differences in the Cyt c protein amounts between the age-matched AL and CR groups at 28 as well as at 32 months. The age-related decrease in Cyt c amounts in the two CR groups showed a trend similar to that observed in the AL-28 and AL-32 groups with a more relevant decrease in the CR-28 animals partially prevented in the older CR-32 rats.

### 2.2. Evaluation of Age and CR Effects on mtDNA Repair Enzymes

**Figure 4:**
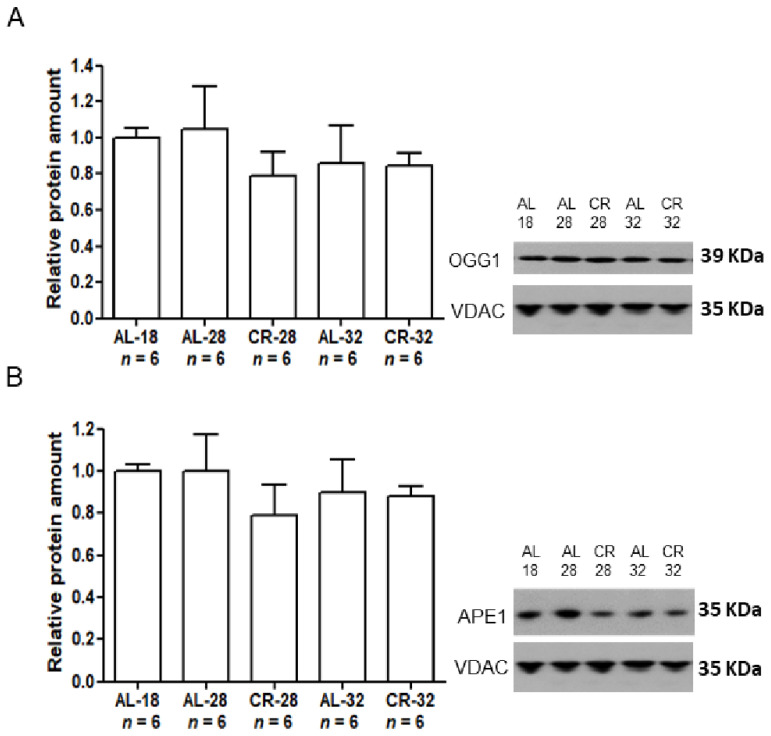
Amounts of OGG1 and APE1 proteins in isolated liver mitochondria from AL-18, AL-28, CR-28, AL-32, and CR-32-month-old rats. (**A**). In the inset, a representative Western blot was carried out in one rat from each of the assayed groups. Immunoreactive bands, from top to bottom represent, respectively, the signals from OGG1 and VDAC. The histogram shows the quantification of the intensity of the bands of OGG1 normalized to the intensity of VDAC. (**B**). In the inset, a representative Western blot was carried out in one rat from each of the assayed groups. Immunoreactive bands, from top to bottom, represent, respectively, the signals from APE1 and VDAC. The histogram shows the quantification of the intensity of the bands of APE1 normalized to the intensity of VDAC. (**A**,**B**) Data represent the results from triplicated Western blot experiments and were analyzed using the One-way ANOVA test and Tukey’s multiple comparison test. Bars represent the mean values and SD for the five experimental groups. Data were normalized against the value of the AL-18 rats, fixed as 1. *n*: Number of analyzed animals.

Since aging has been largely shown to be associated with the accumulation of damage to mtDNA, we measured, by Western blot experiments, in isolated liver mitochondria the expression of two major mtDNA repair enzymes, namely the 8-oxoG DNA glycosylase/apurinic or apyrimidinic (AP) lyase (OGG1) and apurinic/apyrimidinic endonuclease 1 (APE1), both involved in the base excision repair (BER) which is the prevalent mtDNA repair mechanism (Figure 4).

The intra-mitochondrial protein amounts of both enzymes had similar trends as for age and CR effects. In particular, OGG1 and APE1 did not show any statistically significant age-related change in both the AL and CR groups suggesting no age effect at all. Unexpectedly, also CR did not induce any significant effect on OGG1 and APE1 amounts in the CR-28 group in comparison with the age-matched AL animals, as well as between the 32-month-old groups.

### 2.3. Evaluation of Age and CR Effects on Mitochondrial Dynamics

**Figure 5:**
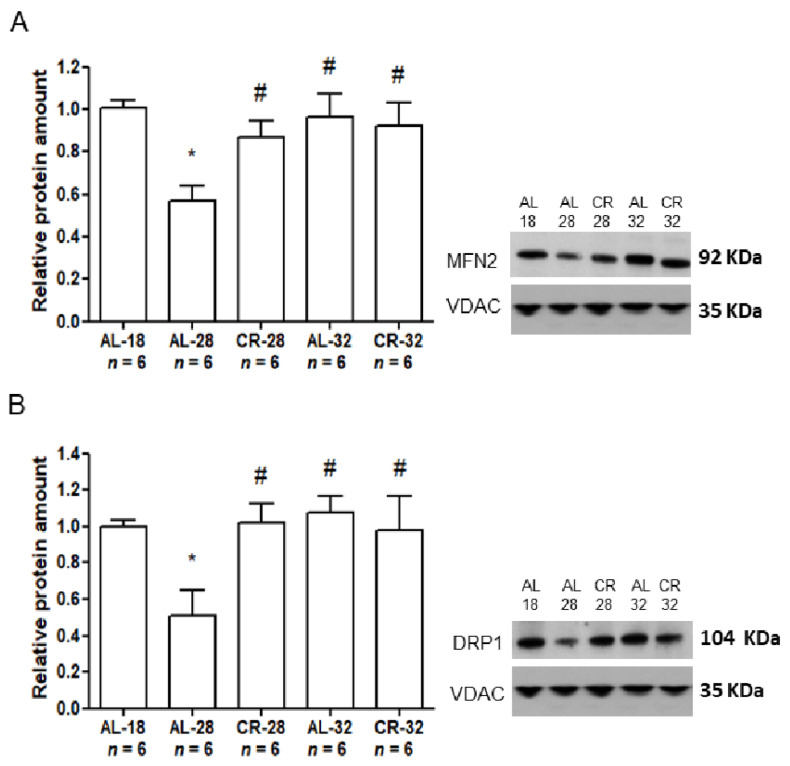
Amounts of MFN2 and DRP1 proteins in isolated liver mitochondria from AL-18, AL-28, CR-28, AL-32, and CR-32-month-old rats. (**A**). In the inset, a representative Western blot was carried out in one rat from each of the assayed groups. Immunoreactive bands, from top to bottom, represent, respectively, the signals from MFN2 and VDAC. The histogram shows the quantification of the intensity of the bands of MFN2 normalized to the intensity of VDAC. (**B**). In the inset, a representative Western blot was carried out in one rat from each of the assayed groups. Immunoreactive bands, from top to bottom represent, respectively, the signals from DRP1 and VDAC. The histogram shows the quantification of the intensity of the bands of DRP1 normalized to the intensity of VDAC. (**A**,**B**) Data represent the results from triplicate Western blot experiments and were analyzed using the One-way ANOVA test and Tukey’s multiple comparison test. Bars represent the mean values and SD for the five experimental groups. Data were normalized against the value of the AL-18 rats, fixed as 1. * *p* < 0.05 versus AL-18 rats, # *p* < 0.05 versus AL-28 rats. *n*: Number of analyzed animals.

Having previously reported a marked age effect on the expression of proteins involved in fusion (MFN2) and fission (DRP1) [16], we evaluated here the effects of CR in a long time span on these markers. Therefore, the protein amounts of MFN2 and DRP1 were determined, by Western blot experiments, in isolated liver mitochondria from all rat groups (Figure 5).

**Figure 6:**
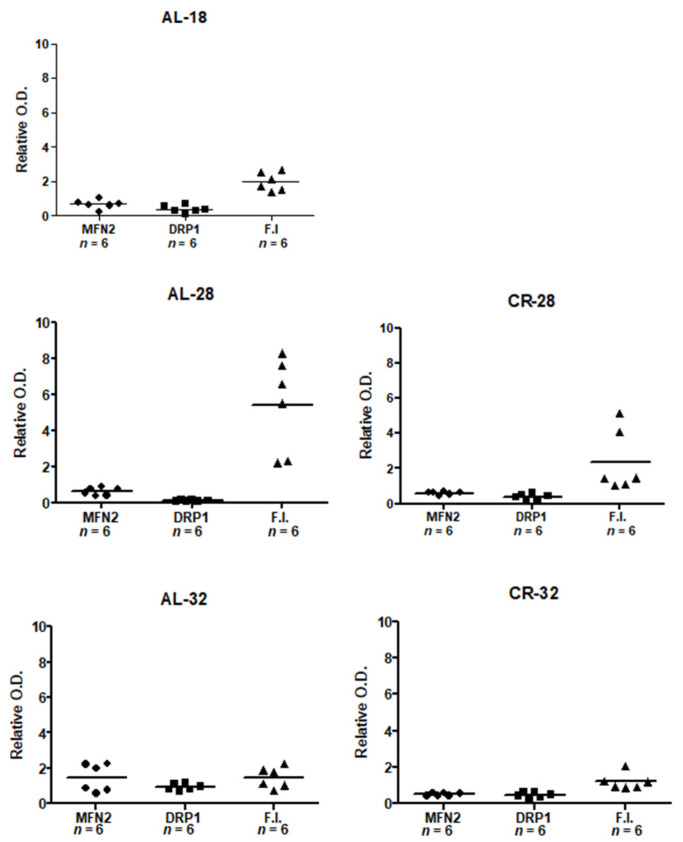
Scatter plots for values of MFN2 and DRP1 protein amounts and of the fusion index (FI) of all the assayed animals. As for MFN2 and DRP1, symbols show the quantification of the intensity of immunoreactive bands of MFN2 or DRP1 normalized to the intensity of VDAC in isolated mitochondria from each rat. All data represent the results from triplicate Western blot experiments. FI was calculated from the ratio of MFN2 and DRP1 intra-mitochondrial protein amounts for each of the assayed animals. The horizontal line represents the mean value. *n*: Number of analyzed animals.

We found a statistically significant age-related decrease (−43%) in MFN2 in the AL-28 rats compared to the AL-18 controls. Such decrease was prevented in the AL-32 rats showing a complex age effect, similar to that reported for Cyt c. However, different from Cyt c, CR had a very positive effect on the 28-month-old animals, preventing in the CR-28 rats the age-related decrease of the AL-28 animals and leading to a significant marked increase (+52%) in MFN2 compared to the AL age-matched counterpart. Furthermore, the MFN2 amount in the CR-28 rats was not different from the value in the AL-18 controls, indicating an efficacious prevention of the age-related effect. No significant difference was found between the AL-32 and CR-32 groups, thus indicating a limited effect of CR in the oldest animals. Moreover, no age-related effect was found for MFN2 in the AL-32 group compared to the AL-18 counterpart. Similarly, the amounts of DRP1 protein showed a marked age-related decrease (−49%) in AL-28 rats compared to the AL-18 group which was prevented by CR in the 28-month-old animals. Indeed, the CR-28 group presented a positive difference (+50%) in comparison with the AL-28 one, demonstrating a strong CR effect. However, the absence of a difference between AL-32 and CR-32 as for the DRP1 protein amounts further indicated a limited efficacy of CR in extended aging. Again, there was no evident age effect also for the DRP1 amounts passing from the AL-18 group to the AL-32 one. Then, we determined the values of fusion index (FI) as the ratio between MFN2/DRP1 for each of the examined rats (Figure 6).

The FI values in the AL-18 group covered a narrow range spanning from 1.37 to 2.64, whereas in the AL-28 group all values were higher ranging from 2.20 to 8.24. The FI values of the CR-28 group ranged from 0.99 to 5.14 and were quite similar to those of the AL-18 rats. In the AL-32 animals, the FI values ranged from 0.70 to 2.27, thus covering an interval similar to that of the AL-18 group which suggests the absence of a marked age effect. In the CR-32 group, the FI values ranged from 0.87 to 2.02 and were comparable to those of the AL-32 animals, thus supporting the idea that extended aging (32 months) may be less sensitive to CR than aging (28 months).

**Figure 7:**
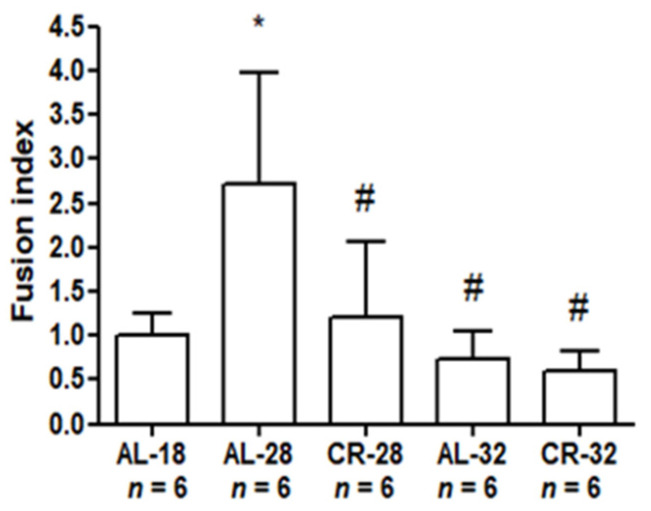
Fusion index (FI) values in isolated liver mitochondria from AL-18, AL-28, CR-28, AL-32, and CR-32-month-old rats. Bars represent the mean values and SD of the ratio, calculated for each group of rats from the individual values in Figure 6. Comparisons were made with respect to the value of the AL-18 rats, fixed as 1. * *p* < 0.05 versus AL-18 rats; # *p*< 0.05 versus AL-28 rats. *n*: Number of analyzed animals.

The statistical analysis of the FI values from all the groups is presented in Figure 7.

In the comparison between AL-18 and AL-28 groups, the higher FI value of the latter indicated an age-related alteration of dynamic balance. As for the CR-treated rats, the value of the CR-28 group was not different from that of the AL-18 counterpart and supported the diet efficacy in the comparison with the age-matched AL-fed counterparts. Analyzing the AL-32 and the CR-32 values of FI the absence of a significant difference between them was evident, reinforcing the idea of a reduced efficacy of the diet in the animals naturally reaching extended aging. Furthermore, the FI values of both 32-month-old groups were not different from the AL-18 counterpart, supporting the absence of a marked age effect in the oldest animals.

### 2.4. Effect of Age and CR on mtDNA Content and Damage

**Figure 8:**
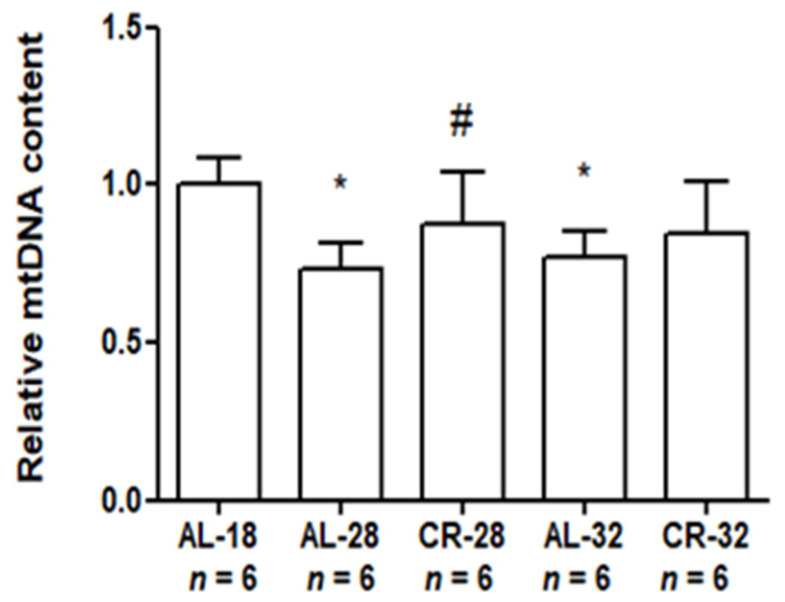
Relative content of mitochondrial DNA (mtDNA) in liver from AL-18, AL-28, CR-28, AL-32, and CR-32-month-old rats. Bars represent data obtained from two independent experiments conducted in triplicate and analyzed using the One-way ANOVA test and Tukey’s multiple comparison test. In the graphical representation, data have been normalized against the value of the AL-18 rats, fixed as 1, and shown as the mean value and SD. * *p* < 0.05 versus AL-18 rats; # *p* < 0.05 versus AL-28 rats. *n*: Number of analyzed animals.

Since mitochondrial dynamics is closely related to the maintenance of mtDNA [16,18], we determined the mtDNA content in all the rats by the quantitative polymerase chain reaction (qPCR) (Figure 8).

An age-related decrease in the mtDNA content was found in the AL-28 (−27%) and the AL-32 rats (−23%) compared with the AL-18 animals, whereas no statistical difference was present between the AL-28 and the AL-32 groups. CR was very effective in preventing the age-related decrease in the mtDNA content only at 28 months leading to an increased (+19%) value in the CR-28 group compared to the AL-fed age-matched counterpart. Furthermore, there was no statistical difference between the AL-18 value and that of the CR-28 group. As seen for other markers in the present work, CR was not so efficacious at 32 months since there was no difference between the AL-32 value and that of the CR-32 counterpart.

**Figure 9:**
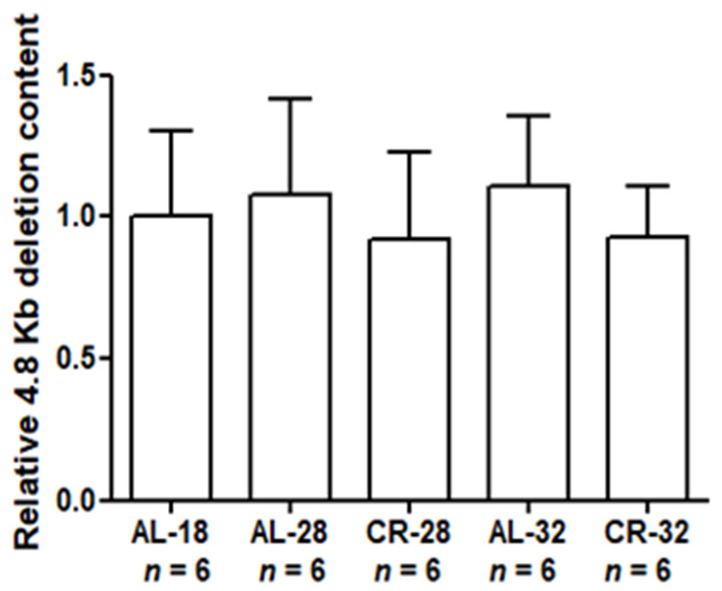
Relative content of the 4.8 Kb deletion in liver from AL-18, AL-28, CR-28, AL-32, and CR-32-month-old rats. Bars represent data obtained from two independent experiments conducted in triplicate and analyzed using the One-way ANOVA test and Tukey’s multiple comparison test. In the graphical representation, data have been normalized against the value of the AL-18 rats, fixed as 1, and shown as the mean value and SD. *n*: Number of analyzed animals.

In order to deepen the analysis at the mtDNA damage level, we measured the relative content of the 4.8 Kb “common deletion” by qPCR (Figure 9).

No significant changes were found with age nor with CR in the relative content of the 4.8 Kb deletion in all the tested groups.

**Figure 10:**
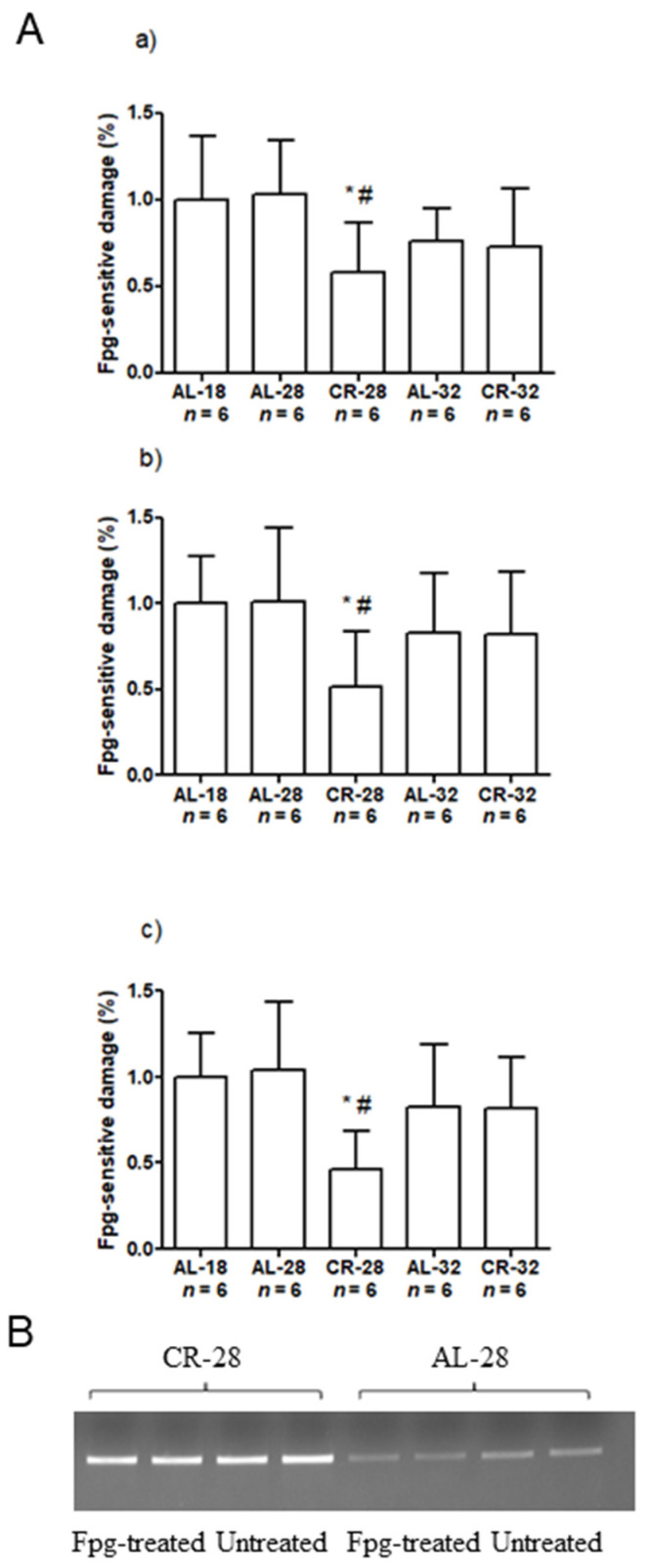
Incidence of oxidized purines damage at the D-loop, Ori-L, and ND1/ND2 mtDNA regions in liver from AL-18, AL-28, CR-28, AL-32, and CR-32-month-old rats. (**A**). Bars represent data obtained from two independent experiments conducted in triplicate and analyzed using the One-way ANOVA test and Tukey’s multiple comparison test. In the graphical representation, data have been normalized against the value of the AL-18 rats, fixed as 1, and shown as the mean value and SD. * *p* < 0.05 versus AL-18 rats; # *p* < 0.05 versus AL-28 rats. *n*: Number of analyzed animals. (**a**) D-loop; (**b**) Ori-L; (**c**) ND1/ND2. (**B**). Representative gel of formamidopyrimidine DNA glycosylase (Fpg)-treated and untreated total DNA from one CR-28 and one AL-28 rat; 5 ng total DNA were amplified using the D-loop primers. An aliquot of each PCR amplification was loaded onto agarose ethidium bromide-stained gel and analyzed for band intensities.

Finally, we screened three specific regions of mtDNA for the incidence of oxidized purines, mainly 8-oxo-deoxyguanosine (8-oxodG), using the oxidized purines-sensitive enzyme formamidopyrimidine DNA glycosylase (Fpg). The three regions encompassed, respectively, the displacement loop (D-loop), the origin of the Light-strand replication (Ori-L), and parts of both the NADH dehydrogenase (ND) subunit 1 and 2 (ND1/ND2) (Figure 10).

As shown in Figure 10, the percentage of 8-oxodG incidence was significantly different between the rat groups in each analyzed region, although with a similar pattern. The post-hoc test revealed the significance of the reduced presence of 8-oxodG in CR-28 rats as compared with both AL-18 and AL-28 groups (−42% CR-28 vs. AL-18 and −43% CR-28 vs. AL-28 at the D-loop; −48% CR-28 vs. AL-18 and −49% CR-28 vs. AL-28 at the Ori-L; −54% CR-28 vs. AL-18 and −56% CR-28 vs. AL-28 at ND1/ND2). CR showed to be effective at preventing the mtDNA oxidative damage up to 28 months, while the incidence of the same damage was not different between AL-32 and CR-32 rats. Oxidized purines were detected already in AL-18 rats without showing an age-related increase in the AL-28 animals. The comparison between the AL-32 group and the CR-32 one did not show a significant difference. Considering the specific regions’ values it is interesting to highlight that the D-loop incidence was the highest among the values from the tested regions in each group, thus supporting previous findings indicating this region as a hotspot for oxidative damage [17,19,20,21].

All the findings reported on the CR efficacy in the age-matched groups were summarized in Table 1.

## 3. Discussion

In the long-lasting search for approaches able to counteract the progressive decline of aging, CR has gained a paramount relevance due to its positive effects, reported in a wide series of organisms ranging from yeast through man [22]. CR has been demonstrated to affect a large number of molecular pathways. Among these, some are particularly effective in the modulation of mitochondrial biogenesis and activity, as those sensing nutrients and/or energy levels such as the AMP-dependent kinase (AMPK) and sirtuins, in which all cooperate to the CR-induced reprogramming of mitochondrial metabolism [23]. By improving the oxidative metabolism, CR reduces the mitochondrial production of ROS and the molecular damages induced by the age-related increase of these reactive species [8,9,10]. Although several studies investigated the effects of CR at the mitochondrial level [3,13,14,15,24], a comprehensive work on the effects of this nutritional intervention on organelles is still missing. Therefore, this study sought to test the existence of boundaries to the CRs efficacy in a large set of mitochondrial markers. In particular, we focused on the comparison between the effects induced by this dietary regimen in aged (28-month-old) and extremely aged (32-month-old) rats to verify whether an interplay exists between CR and the progression of aging. The analysis of the results revealed a complex picture as for the efficacy of CR on mitochondrial biogenesis and mtDNA damage, which has been summarized in Table 1.

### 3.1. Mitochondrial Markers in 28-Month-Old Rats

A large number of mitochondrial markers was positively affected by CR in 28-month-old rats. Namely, here were reported age-related decreases in the citrate synthase activity, in protein amounts of TFAM, MFN2, and DRP1 and in the mtDNA content comparing the AL-18 control group with the AL-28 one. Such changes were all prevented by CR in 28-month-old rats (CR-28). These results support the anti-aging effect of CR up to this age in rat liver. Similarly, the decrease in the incidence of oxidized purines at all the three assayed mtDNA regions, induced by CR, further reinforces this view. Of note, this oxidative damage was already present in the AL-18 animals with no age-related increase in the AL-28 rats suggesting that its appearance may be identified as an early marker of aging. A particular attention needs to be placed on the five mitochondrial markers that were not affected by CR in 28-month-old rats namely the protein amounts of LonP1, Cyt c, OGG1, and APE1 and the 4.8 Kb deletion content. In fact, as for LonP1 and Cyt c, CR did not prevent the age-related decrease found in the AL-28 animals, whereas the protein amounts of OGG1 and APE1 as well as the 4.8 Kb deletion content were not affected by age nor by CR. LonP1 is responsible for the degradation of exhausted TFAM [25,26,27] and has been reported to decrease with aging [28] in rat liver [29] up to 27 months of age. We previously suggested that such aging-decreased expression of LonP1 [16] might be related to its activity in the removal of TFAM unbound to mtDNA [26]. As a possible explanation for the inefficacy of CR to prevent the age-related decrease in LonP1 expression, despite the restored TFAM expression and TFAM-binding to mtDNA specific regions (present data and Picca et al. [15]), is the CR-mediated activation of Sirt3 [30] that might have influenced also the protein amount of LonP1 via the induced deacetylation. A different hypothesis can be proposed for explaining the inefficacy of CR to prevent the age-related decrease in the amount of Cyt c. This protein is the major trigger of the mitochondrial apoptotic pathway through its release to the cytosol, thus its decreased intra-mitochondrial amount in the AL-28 rats with respect to the AL-18 value indicated an increase in the intrinsic apoptotic process with aging [16,31]. Furthermore, as for LonP1, the Cyt c amount in the CR-28 rats was still significantly lower than the value in the AL-18 animals, indicating that CR did not substantially affect the final regulation of Cyt c expression/release to cytosol and, presumably, nor the age-related increase in mitochondrial apoptotic pathway. This is in agreement with the previously reported promotion of apoptosis mediated by CR [23]. Since the apoptotic pathway can be triggered also by the accumulation of DNA damage [32,33], we determined the protein expression of two major mitochondrial BER enzymes namely OGG1 and APE1. The protein amounts of both were not affected by aging and CR passing from 18 to 28 months of age. A possible explanation is that the expression of the two BER enzymes was already increased and sufficient in the AL-18 rats to counteract the age-related increased damage to mtDNA. Indeed, an age-related increase in the activity of mitochondrial OGG1 [34,35] and mitochondrial APE1 [36] has been reported in the liver of rodents passing from 3–6-month-old animals to 20–23-month-old ones, likely induced to efficiently counteract the age-related increase in mtDNA damage. All this further supports our hypothesis that the mtDNA damages, repaired by OGG1 and APE1, appeared very early in rodents’ lifetime (they were already present in the AL-18 rats) and induced, through a mitochondrion-nucleus retrograde communication, an early increase in expression of the BER enzymes. As for the absence of a CR effect on OGG1 and APE1, we suggest that the CR-reduced presence of ROS and related oxidative damages was efficaciously counteracted by an expression of both enzymes not different from that of the age-matched AL-animals. As for the 4.8 Kb deletion content, there was no age-related increase passing from the AL-18 animals to the AL-28 ones as if also this mtDNA damage appeared quite early in rats’ lifetime and its age-related increase could have been verified only in comparison with young animals, as reported in other studies [19,37,38,39]. The inefficacy of CR to significantly decrease the 4.8 Kb deletion content in the CR-28 rats in comparison with the AL-28 and the AL-18 animals further indicates that the long-term CR induced a marked decrease in the presence of ROS in liver. This might have led to a sort of steady-state level of the 4.8 Kb deletion, not different from that of the AL-18 counterpart and compatible with the respective mtDNA content. Although the mtDNA repair was not reduced by aging, the age-related decrease in the mtDNA content was still present when passing from the AL-18 rats to the AL-28 ones, as previously described in different tissues of aged rodents [3,15,19,39,40,41,42]. This may be explained by the age-related increased binding of TFAM at specific mtDNA regions that were also damage hotspots [19]. This hypothesis would thus reconcile the age-related mtDNA loss with the unchanged expression and activity of the repair enzymes. In further support to this hypothesis is the absence of mtDNA loss in the CR-28 rats. Indeed, we previously demonstrated that CR prevented the age-related increase in TFAM-binding at specific mtDNA regions [15]. Therefore, according to the present and our previous data, the modulation of TFAM-binding may be among the molecular mechanisms involved in the aging- and CR-mediated regulation of mitochondrial biogenesis. This regulation by aging and CR has also been shown to be pursued through the balance of mitochondrial dynamics which is affected by aging [43] and leads to the prevalence of fusion over fission [44,45]. Therefore, we calculated the fusion index (FI) in all assayed animals (Figure 6) and found values that confirmed and expanded our previous results [16]. Some novel indications can be gathered by the present study, namely in the AL-18 rats the FI values covered a small range, while in the AL-28 ones the FI range was much larger, with a marked age-related increase in the individual values, in the CR-28 rats the small range of values indicated a positive effect of CR in the induction of fission, with a generalized increase in the DRP1 individual amounts in comparison with those from the AL-28 group. While an efficient fission might remove the damaged organelles, a similar entity fusion might ensure the diffusion of damaged macromolecules in the mitochondrial network, thus diluting their negative effects [46]. Therefore, the CR-mediated restoration of the finely tuned-up balance of mitochondrial dynamics might induce positive consequences on the modulation of mitochondrial biogenesis, acting synergistically with the mtDNA damage retrograde communication. This is in line with the existing literature reporting that the nutrient status and metabolic alterations can modulate the balance of mitochondrial dynamics [47] and, thus, affect also the organelle biogenesis. In particular, the ROS-sensitive regulation of mitochondrial biogenesis can face only limited increases of ROS through the increase in organelles number [48] and the CR-reduced presence of ROS [15] might prevent overcoming of a threshold-like level that might lead to mitochondrial loss. The CR efficacy in decreasing the presence of ROS was proven in the present study by the reduced accumulation of oxidized purines at the mtDNA analyzed regions in the CR-28 rats that supports an overall improvement of mitochondrial metabolism, able to counteract the age-related decline. Indeed, the CR-reduced presence of ROS has been widely demonstrated also at the level of mitochondrial proteins and lipids. In particular, CR has been shown to decrease oxidative modifications of mitochondrial proteins in rat heart [49] and to modify the saturation/unsaturation index in mitochondrial membranes preventing oxidative damage and maintaining membrane fluidity [8]. Furthermore, CR has been demonstrated to decrease the production of ROS by mitochondrial respiratory complexes in both murine skeletal muscle and liver [8]. The CR beneficial effects at the ROS level also include an improved neutralization of ROS and an increased repair of ROS-damaged molecules [10]. In addition to this CR effect, changes in mitochondrial functionality are modulated by the activation of CR-sensitive AMPK and sirtuins that regulate the activity of the transcription factors peroxisome proliferator-activated receptor gamma coactivator 1-alpha (PGC-1α), and forkhead box (FoxO), with the latter involved in biogenesis, oxidative metabolism, and turnover of the mitochondria [23]. Thus, the CR-mediated positive influence on mitochondrial metabolism should interact with other pathways to modulate the final trigger of mitochondrial biogenesis, according to a model of retrograde communication to the nucleus [50].

### 3.2. Mitochondrial Markers in 32-Month-Old Rats

As for the comparison between AL-32 and CR-32 rats, our data show a generalized absence of significant differences. This may suggest an age limitation to the efficacy of CR in counteracting the age-related decline when passing from 28 to 32 months. With regards to the age effect, though, different conclusions can be driven depending on the analyzed marker. In fact, in both 32-month-old groups, the age-related decrease in the citrate synthase activity, amounts of TFAM, LonP1, OGG1, APE1, contents of mtDNA, and of the 4.8 Kb deletion was maintained at a level which was similar to that observed in the AL-28 rats or it was partially/completely prevented as in amounts of Cyt c, MFN2, DRP1, and the incidence of oxidized purines at specific mtDNA regions. These different behaviors may be likely due to the interplay of the various pathways involving the analyzed markers. Another conclusion can be highlighted comparing both 32-month-old groups with the AL-18 and the AL-28 ones as for the MFN2 and DRP1 amounts. In fact, the age-related decrease was not present passing from the AL-18 group to both AL-32 and CR-32 groups for the two dynamics proteins. Considering that in the comparison between the AL-18 group and both the 32-month-old ones as for the other markers there was an age-related decrease similar or smaller in size than that between the AL-18 and AL-28 groups, we can drive some consequences. This may indicate that the age-related alterations of markers may have begun or been already present at 18 months, and then reached their lowest/highest value in the aged, 28-month-old animals with a “pace” which is typical of the aging process. Conversely, in the extremely aged animals (32-month-old), the “pace” of the process appeared slowed since the values lower than or equal to those of the AL-18 counterparts, but not lower/higher than those from the AL-28 ones, were found, despite the longer time span elapsed. The hypothesis of two different paces in the aging process [16] implies that damages, due to different causes, begin to affect physical and metabolic activities during adulthood in each rat. Such damages are efficaciously counteracted by compensatory mechanisms and the damages do not appear openly, coinciding with a stable “middle” age characterized by a successful homeostasis. In this study, we identified as rats aging “fast” those in which the balance between intervening damages and compensatory mechanisms begins to fade later in life. In these animals, constituting the larger fraction of the AL-28 group, damages exceed the threshold compatible with normal activities and an altered condition appears. Conversely, the remaining, smaller part of the examined aging population maintains the homeostatic balance between the damages and repair for a longer time and constitutes the group of animals aging “slowly”, including both AL-32 and CR-32 rats. A similar hypothesis about the different paces of aging has recently been proposed also for humans [51]. Taken as a whole, these results indicate that the extremely aged animals may have reached longevity without being significantly affected by CR and likely due to a genetic predisposition to better cope with the age-related mitochondrial dysfunction, but such hypothesis warrants future research. These novel findings can give an answer to the original and crucial question on the existence of limits to the efficacy of CR, raised by Ingram and de Cabo in their very deep study on this issue [52], allowing the identification of such limit with the age of 28 months for rat liver. Nevertheless, the conclusion that CR or any diet did not elicit beneficial consequences in those rats naturally predisposed to longevity should not affect the overall confidence in efficacy of this nutritional intervention. In fact, CR was still able to convey beneficial effects as it was able to prevent most of the age-related alterations of mitochondrial biogenesis and mtDNA damage in the liver of 28-month-old rats. This further confirms CR as a well-founded strategy to efficiently counteract the aging decline in the general population.

## 4. Materials and Methods

### 4.1. Animals

The study was approved by the Institutional Animal Care and Use Committee at the University of Florida. All procedures were performed in accordance with the National Institute of Health (NIH) guidelines for the care and use of laboratory animals. Liver samples were from Fischer 344 × Brown Norway (F344BNF1) male rats obtained from the National Institute of Aging colony (Indianapolis, IN, USA) and housed individually in a temperature- (20 +/− 2 °C) and light-controlled environment (12-h light/dark cycle) with regular rat chow and water available ad libitum at the Department of Aging and Geriatric Research, Division of Biology of Aging, College of Medicine, University of Florida, Gainesville, FL (USA). Calorie restriction had been initiated at 3.5 months of age (10% restriction), raised to 25% restriction at 3.75 months, and kept at 40% restriction from 4 months until the end of each animal’s life, which is at 28 months (CR-28) or 32 months (CR-32) of age. Calorie-restricted animals were fed the NIH31-NIA fortified diet to ensure that they were not malnourished, whereas ad libitum-fed animals were given the NIH31 rat diet. The animals consisted of the following groups: 18-month-old ad libitum-fed (AL-18, *n* = 6), 28-month-old ad libitum-fed (AL-28, *n* = 6), 28-month-old calorie-restricted (CR-28, *n* = 6), 32-month-old ad libitum-fed (AL-32, *n* = 6), and 32-month-old calorie-restricted (CR-32, *n* = 6) rats. Animals were anesthetized before sacrifice and liver samples were immediately removed, snap-frozen in isopentane cooled by liquid nitrogen, and stored in liquid nitrogen until further analysis.

### 4.2. Determination of Citrate Synthase Activity

Determination of citrate synthase activity was performed as in [17]. Briefly, 80 μg of total proteins purified from liver samples were incubated in the reaction buffer (0.31 mM acetyl-CoA, 100 mM Tris buffer (pH 8.1), 0.25% Triton X-100, 0.1 mM 550-dithio-bis-2-nitrobenzoic acid, and 0.5 mM oxaloacetate (1 mL) at 30 °C.

The citrate synthase activity (μmol × min^−1^ × g tissue^–1^) was spectrophotometrically determined by measuring the rate of production of thionitrobenzoic acid (TNB) at 412 nm.

### 4.3. Western Immunoblotting

Liver mitochondria were isolated in a medium containing 220 mM mannitol, 70 mM sucrose, 20 mM Tris–HCl, 1 mM EDTA, and 5 mM EGTA, pH 7.4, at 4 °C according to [16]. In addition, 10 μg of mitochondrial proteins were used for Western immunoblotting analysis. Anti-TFAM (1:50,000), anti-VDAC (1:50,000, Abcam, Cambridge, UK), anti-OGG1 (1:2500, Abcam, Cambridge, UK), anti-APE1 (1:5000, Abcam, Cambridge, UK), anti-MFN2 (1:5000, Abnova, Taipei, Taiwan), anti-DRP1 (1:2500, Abnova, Taipei, Taiwan), anti-Cyt c (1:500, Pharmingen, San Diego, CA, USA), and anti-Lon Protease (1:10,000) were used as primary antibodies. The antibodies against TFAM and Lon were custom-made and kindly donated, respectively, by Dr. H. Hinagaki (Department of Chemistry, National Industrial Research Institute of Nagoya, Nagoya-shi, Aichi, Japan) and Dr. C. Suzuki (Department of Biochemistry and Molecular Biology, New Jersey Medical School, University of Medicine and Dentistry of New Jersey, Newark, NJ, USA). The proteins were detected by chemiluminescence and immunoreactive bands were quantified using the Image Lab Software (BioRad Laboratories Inc., Hercules, CA, USA) and normalized against VDAC-expression.

### 4.4. Determination of mtDNA and mtDNA 4.8 Kb “Common Deletion” Content

The quantitative real time polymerase chain reaction (qPCR) was used to determine relative contents of mtDNA and mtDNA 4.8 Kb “common deletion”. Reactions were performed via SYBR Green chemistry using 3 ng of total DNA as a template and the following primers: MtDNA for 5′-GGTTCTTACTTCAGGGCCATCA-3′ (nt 15,785–15,806), mtDNA rev 5′-TGATTAGACCCGTTACCATCGA-3′ (nt 15,868–15,847); β-actin for 5′-CCCAGCCATGTACGTAGCCA-3′ (nt 2181–2200), β-actin rev 5′-CGTCTCCGGAGTCCATCAC-3′ (nt 2266–2248); 4.8 del for 5′-AAGGACGAACCTGAGCCCTAATA-3′ (nt 8109–8131), 4.8 del rev 5′-CGAAGTAGATGATGCGTATACTGTA-3′ (nt 13,020–12,996). The mtDNA content relative to the nuclear DNA and 4.8 Kb “common deletion” content relative to mtDNA were determined, as reported in [19].

### 4.5. Analysis of Oxidized Purines

Formamidopyrimidine DNA glycosylase (Fpg) (New England Biolabs, Beverly, MA, USA) digestion of total DNA was used to detect oxidized purines [53].

PCR amplification of the D-loop, Ori-L, and ND1/ND2 regions of mtDNA was conducted using the respective primers pair:

D-loop For 5′-TCTGGTCTTGTAAACCAAAAATGA-3′ (nt 15,302–15,325), D-loop Rev 5′-TGGAATTTTCTGAGGGTAGGC-3′ (nt 16,302–16,282); Ori-L For 5′-AACCAGACCCAAACACGAAA-3′ (nt 4414–4433), Ori-L Rev 5′-CTATTCCTGCTCAGGCTCCA-3′ (nt 5407–5388); ND1 For 5′-AGGACCATTCGCCCTATTCT-3′ (nt 3390–3409), ND1 Rev 5′-CGCCAACAAAGACTGATGAA3′ (nt 4399–4380) on 5 ng of Fpg-treated and untreated total DNA. The cycling conditions were: Pre-incubation of 10 min at 95 °C, followed by 18 cycles of 15 s 95 °C, 15 s 58 °C, and 1 min 72 °C. An aliquot of each PCR amplification was loaded on 1.3% agarose gel. Band intensities of ethidium bromide-stained bands were analyzed by the Image Lab Software (BioRad Laboratories Inc., CA, USA). To improve the graphical visualization, the ratio between Fpg-treated and untreated band intensities was expressed as a percentage of the complement to 100.4.6. statistics

Data are expressed as the mean and standard deviation (SD). The One-way ANOVA with Tukey’s multiple comparison test were used. For all tests, the statistical significance was set at a 5% level. Analyses were run using a specific statistical package (Stata Corp. 2005. Stata Statistical Software: Release. College Station, TX, USA).

## Figures and Tables

**Table 1:** Effect of CR on mitochondrial markers.

Mitochondrial Markers	28 Months (AL vs. CR)	32 Months (AL vs. CR)
Citrate synthase activity	+	−
TFAM	+	−
LonP1	−	−
Cyt c	−	−
OGG1	−	−
APE1	−	−
MFN2	+	−
DRP1	+	−
mtDNA content	+	−
mtDNA 4.8 Kb deletion	−	−
Oxidized purines-specific mt-DNA damage D-loop	+	−
Oxidized purines-specific mt-DNA damage Ori-L	+	−
Oxidized purines-specific mt-DNA damage ND1/ND2	+	−

+: Difference between AL and CR; −: No difference between AL and CR.

## Data Availability

The data that support the findings of this study are available from the corresponding author upon reasonable request.
